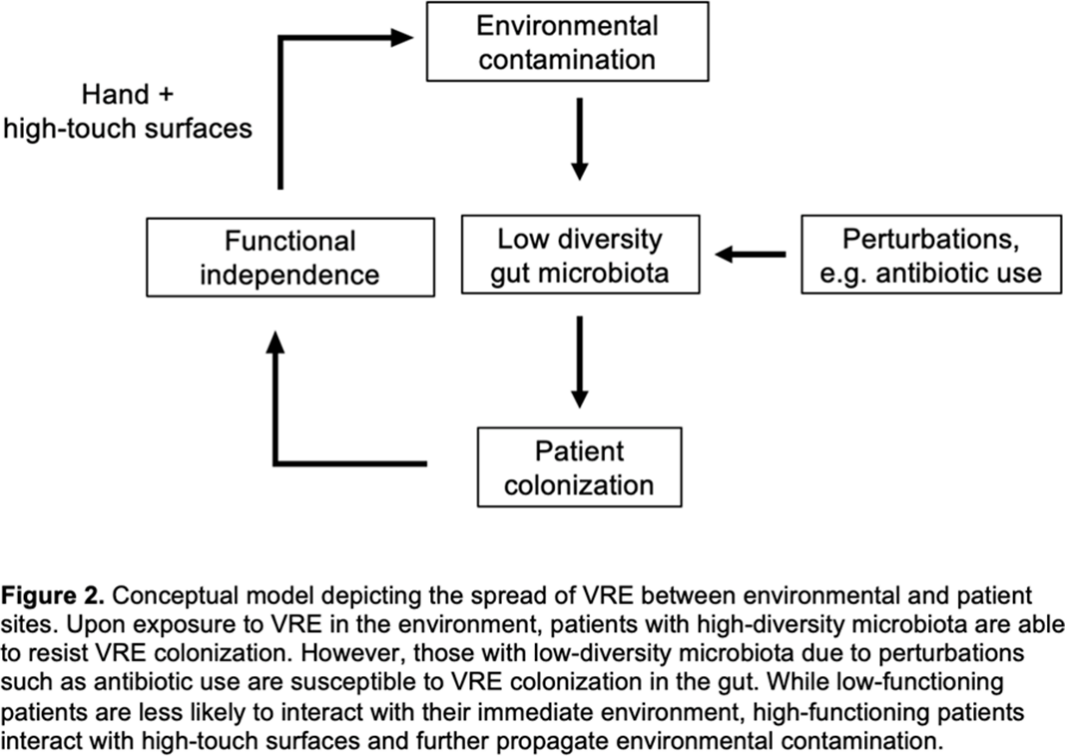# Nursing-Home Patient Functional and Microbiota Status Drive Environmental Contamination with Vancomycin-Resistant Enterococci

**DOI:** 10.1017/ash.2021.132

**Published:** 2021-07-29

**Authors:** Joyce Wang, Betsy Foxman, A. Krishna Rao, Lona Mody, Evan Snitkin

## Abstract

**Background:** Patient colonization and shedding of vancomycin-resistant enterococci (VRE) is a major source of environmental contamination leading to VRE transmission in nursing homes. We hypothesize that we can inform mitigation strategies by identifying patient clinical and microbiota features associated with environmental contamination with VRE. **Methods:** During a 6-month period of active surveillance in 6 Michigan nursing homes, 245 patients (with 806 follow-up visits) were enrolled. Patient clinical data and swabs for VRE were collected from multiple body sites and high-touch environmental surfaces. In total, 316 perirectal swabs were collected from 137 patients for gut microbiota analysis and community status type (CST) assignment based on taxonomic composition. The associations between VRE colonization pattern, gut microbial CST, and patient factors were examined using multivariable generalized estimating equations, adjusting for patient-and facility-level clustering. We used VRE colonization patterns to group study visits: “uncolonized” (patient−/environment−); “environment-only” (patient−/environment+); “patient-only” (patient+/environment−); “both” (patient+/environment+). **Results:** Across all study visits, VRE colonization on patient hand and groin/perirectal area was positively correlated with VRE contamination of high-touch environmental surfaces, suggesting direct transfer of VRE between patient and environment via patient hands (Figure 1A). We next set out to identify patient factors associated with patient colonization and environmental contamination. At baseline, while patients in the “both” group had anticipated risk factors such as longer prior hospitalization and more frequent broad-spectrum antibiotic use, they were unexpectedly younger than “uncolonized” patients and had similar functional status. This last feature contrasted with the “patient-only” group, characterized by higher urinary catheter use and higher functional dependence, suggestive of lower functional dependence facilitating patient contamination of their environment. No clinical features distinguished “uncolonized” and “environment-only” patients (Table 1). Lastly, in multivariable analyses, we determined the contribution of patient functional status and gut microbiota features to environmental contamination. Low-diversity CST, characterized by reduced anaerobic taxa, was weakly associated with “patient-only” and significantly associated with “both.” Notably, high functional dependence was significantly associated with “environment-only” and “patient-only” but not “both,” indicating high-functioning patients with disrupted gut microbiota as drivers of environmental contamination (Figure 1B). **Conclusions:** Our findings suggest that antimicrobial exposure disrupts patient gut microbiota, a significant mediator of colonization dynamics between patients and their environment, and that high-functioning patients may be more likely to spread VRE between their body sites and high-touch environmental surfaces (Figure 2). These findings highlight both antibiotic stewardship and patient hand hygiene as important targets for interrupting transmission mediated by environmental contamination.

**Funding:** No

**Disclosures:** None

**Figure 1.**
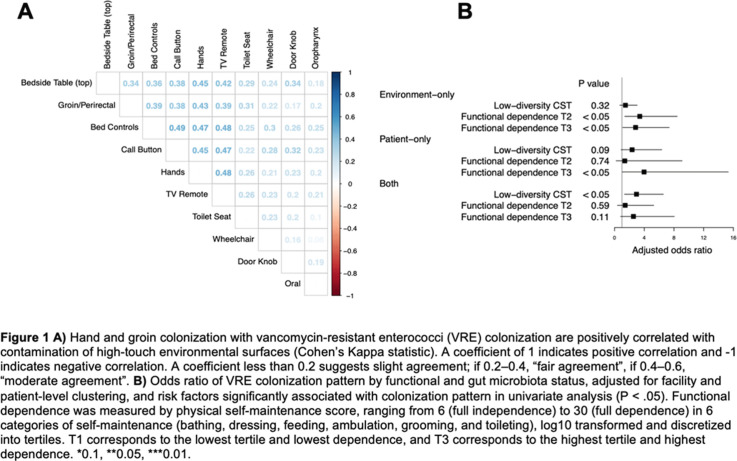

**Table 1.**
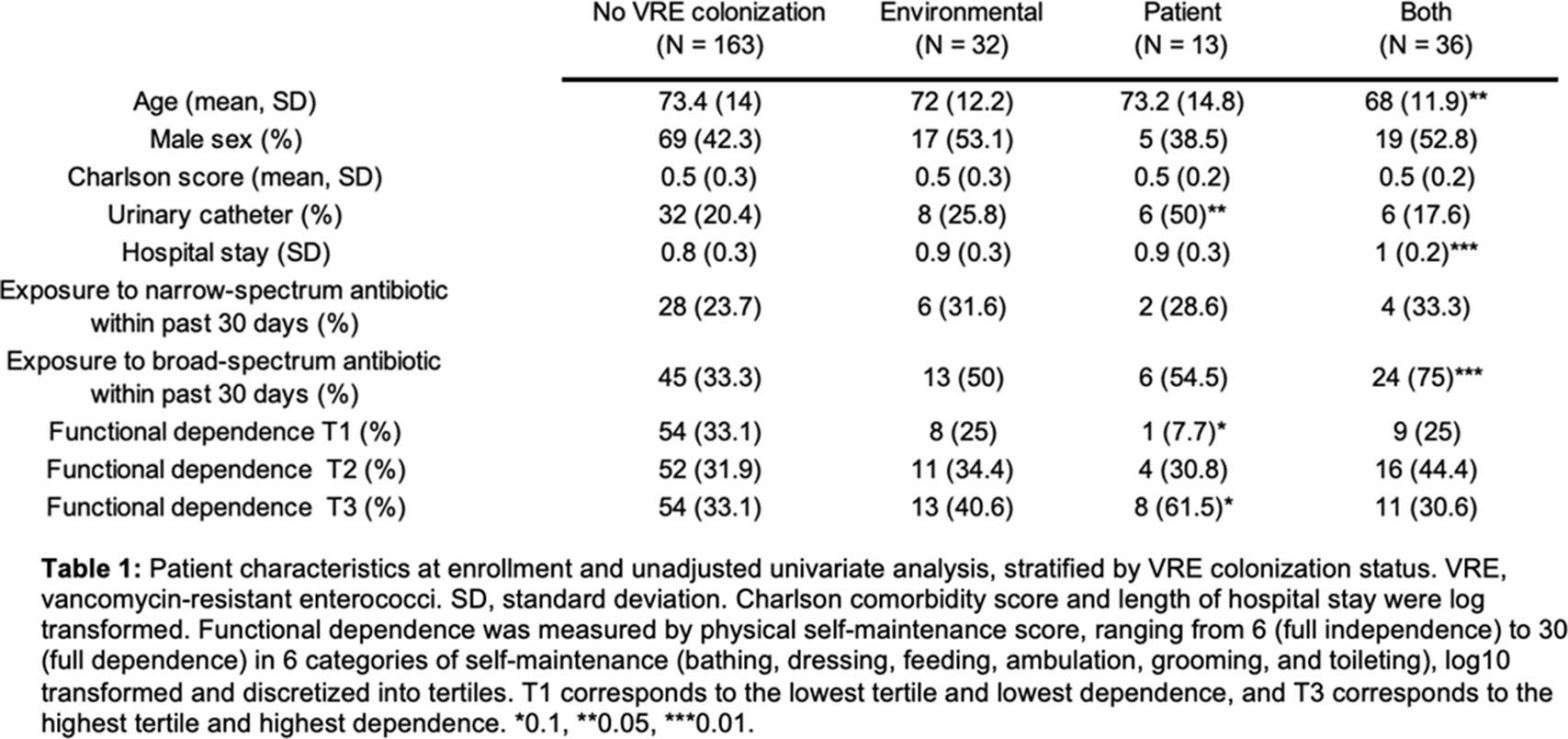

**Figure 2.**